# DEEP Phaser: A Deep Learning Tandem Vision Transformer for Fully Automated NMR Phase Correction

**DOI:** 10.1021/acs.jpclett.6c00770

**Published:** 2026-05-04

**Authors:** Da-Wei Li, Lei Bruschweiler-Li, Kyungsuh Lee, Rafael Brüschweiler

**Affiliations:** † Department of Chemistry and Biochemistry, The Ohio State University, Columbus, Ohio 43210, United States; ‡ Department of Biological Chemistry and Pharmacology, The Ohio State University, Columbus, Ohio 43210, United States

## Abstract

Although phase correction is one of the most routine steps in NMR data processing, even the best available automated approaches often require manual adjustments by human experts. A deep learning-based phase correction algorithm is presented as a tandem vision transformer artificial neural network. It has been trained on a large set of synthetic solution-NMR like spectra and determines the zeroth- and first-order phase correction based on the entire input spectrum and achieves very high phasing accuracy for a broad range of experimental spectra without requiring any further manual adjustments. The new method, called DEEP Phaser, is demonstrated for a variety of different real-world solution ^1^H 1D NMR spectra, from small molecules and their complex mixtures to biomacromolecules, and is available as free software and as a public web server.

## Graphical abstract

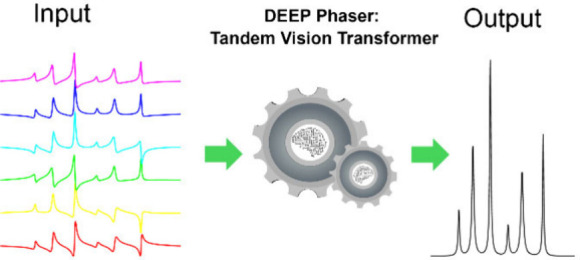

Due to its high versatility, nuclear magnetic resonance (NMR) spectroscopy has found powerful applications in a broad range of scientific disciplines. Since its introduction in the mid-1960s,^1^ time-domain Fourier transform NMR^2^ has become the method of choice by detecting the NMR signal as a free induction decay (FID) in a resonant NMR coil in phase-sensitive quadrature mode followed by complex Fourier transformation. The phase-sensitive nature of the spectrum can be optimally utilized only when it is in 100%-pure absorption mode, which is achieved by a *phase correction* of the complex spectrum. Pure absorption spectra are not only aesthetically pleasing, but they also provide spectroscopic advantages over dispersive or mixed-phase spectra in terms of their narrower resonances, thereby minimizing peak overlaps, along with the positivity of the signals for a fully quantitative analysis based on accurate peak positions and peak volumes.

The conversion of the complex FID *s*(*t*) to the final spectrum *S*(ω) involves several routine processing steps. They include zero-filling, apodization, fast Fourier transformation (FFT), and phase correction. Additional steps may or may not be required depending on the type of experiment and the application, such as linear prediction, NUS reconstruction, or baseline correction, which all may introduce some spectral artifacts and are not further discussed here. Phase correction is the process of changing the phase φ (in units of radian) at each point of the spectrum as a function of the frequency offset ω by multiplying each complex spectral point by a phase factor:

1 $$e^{i\varphi_{0}+ia\omega }$$

where φ_0_ is the frequency-independent “zeroth-order” phase correction and *a*ω is the frequency-dependent “first-order” phase correction where prefactor *a* is a constant corresponding to an effective delay between the radio frequency (rf) detection pulse and the first time point sampled in the FID. In the following we will refer to φ_0_ as PH0 and the first order term is characterized by PH1, which is the total phase change over the entire spectral width. Hence, the phase correction for the *k* ^th^ spectral data point *S*_*k*_ can be expressed as

2 $${PH}_{k}=PH0+PH1\frac{k}{N}$$

where *N* is the total number of complex points with *k* = 1, ..., *N*. Once the phase correction has been applied, the real part of *S* is the absorption spectrum and the imaginary dispersive part can be discarded. Although in eqs 1 and 2, all phases are in units of radian, they are usually discussed in units of degrees.

Since phase correction requires the optimization of only two (real) parameters for the entire spectrum, PH0 and PH1, it is tempting to assume that full automation of this process is straightforward. Although a host of different automated methods have been proposed in the literature (*vide infra*), their phasing accuracy is typically limited to an average accuracy of several degrees or more, also depending on the spectrum. This may be acceptable for some applications but falls short for fully quantitative analysis. In such cases, expert human intervention is required to achieve optimal phase correction. It is therefore desirable to have a general, fully automated phase correction method that meets the highest standards and is comparable to the results obtained by human experts.

Automated phase correction methods can be traced back to the early days of Fourier transform NMR^3–6^ and have been reviewed more recently.^7^ Proposed methods include the integrals of the absorption vs the dispersion signals, the ratio of maximum to minimum signal excursion,^3–6^ absorption vs dispersion plots,^8^ symmetry of resolved absorptive line shapes,^5^ the total peak area of individual peaks,^9^ the flatness of the baseline at the base of sharp peaks,^3–6^ and other methods that combine phase with baseline correction approaches,^10^ although *ad hoc* baseline corrections may affect the quantitative nature of the spectrum. An iterative optimization approach employing multiple objective functions was proposed for tracking NMR spectra containing dense spectral regions with overlapping peaks.^11^ Another strategy for automatic phasing derives from pattern recognition strategies, such as the identification of spectral regions with individual resonances dipping below the baseline for each individual peak.^12^ Entropy minimization methods leverage the premise that a correctly phased spectrum ought to possess a minimal set of features.^13^ The phase correction method “Automics” aims at achieving maximum flatness at both ends of the spectrum.^14^

In recent years, the advent of deep artificial neural networks has provided new opportunities for machine-learning based tools for the automated analysis of NMR spectra^15,16^ For example, the recently introduced DEEP Picker and Voigt Fitter tools perform convolutional neural network (CNN) based peak picking and fitting for fully automated quantitative NMR spectral deconvolution, requiring well-phased spectra as input.^17,18^ Recently, a machine-learning approach using a classical convolutional neural network demonstrates good phase correction performance when combined with residual baseline removal, which was implemented in Bruker’s Topspin software. Moreover, the PD-RAN approach^19^ adapts residual attention networks to NMR spectroscopy by reshaping 1D spectra into 2D images to predict zero and first order phase correction parameters directly. These latest methods have a reasonable success rate when applied to a variety of spectra often requiring additional manual tweaking for obtaining optimal results. Here, we introduce an automated phase correction method that utilizes the combination of a CNN and an attention neural network model^20^ and which is demonstrated for a selection of real-world solution NMR spectra acquired at different magnetic field strengths. This method complements our COLMAR suite^21,22^ for automated 1D NMR applications and is implemented in the COLMARvista^23^ spectral processing and visualization software. The automated phase correction method, called DEEP Phaser, further streamlines NMR data analysis representing a significant step toward fully automated, reproducible, and accurate spectral processing and analysis.

Figure 1 shows a single synthetic Voigt-shaped NMR peak with and without a phase error. The phase error of 0.5° is manifested as a slightly asymmetric peak shape (Panel A) with a characteristic sharp dip (“foot”) on one side of the peak (right) followed by gradual recovery of the spectral profile for large offsets toward zero (Panel B). Experienced NMR spectroscopists are adept at recognizing such patterns, even when present only weakly, enabling optimal phase correction by eliminating surrounding dips near peaks across the entire spectrum. Alternatively, machine learning algorithms, such as convolutional neural networks (CNN), offer potentially fully automated solutions due to their excellent pattern recognition capabilities. However, CNNs have limitations in recognizing spectral properties over larger frequency ranges (“long-range correlations”), because of their limited field of view, which hinders the identification of small (<1°) phase errors. As shown in Figure 1B, spectroscopists are used to oftentimes zoom in to <1% of the maximal peak height to examine spectral regions approximately 50 times the peak width away from the peak center for detecting small phase errors. This is essential because small phase errors can be indistinguishable from correctly phased peaks, particularly on large intensity or narrow frequency scales, as shown in Figure 1A. Moreover, global inspection of the spectrum, particularly at both the left and right ends, is necessary to correct potential first-order phase errors.

**Figure 1:**
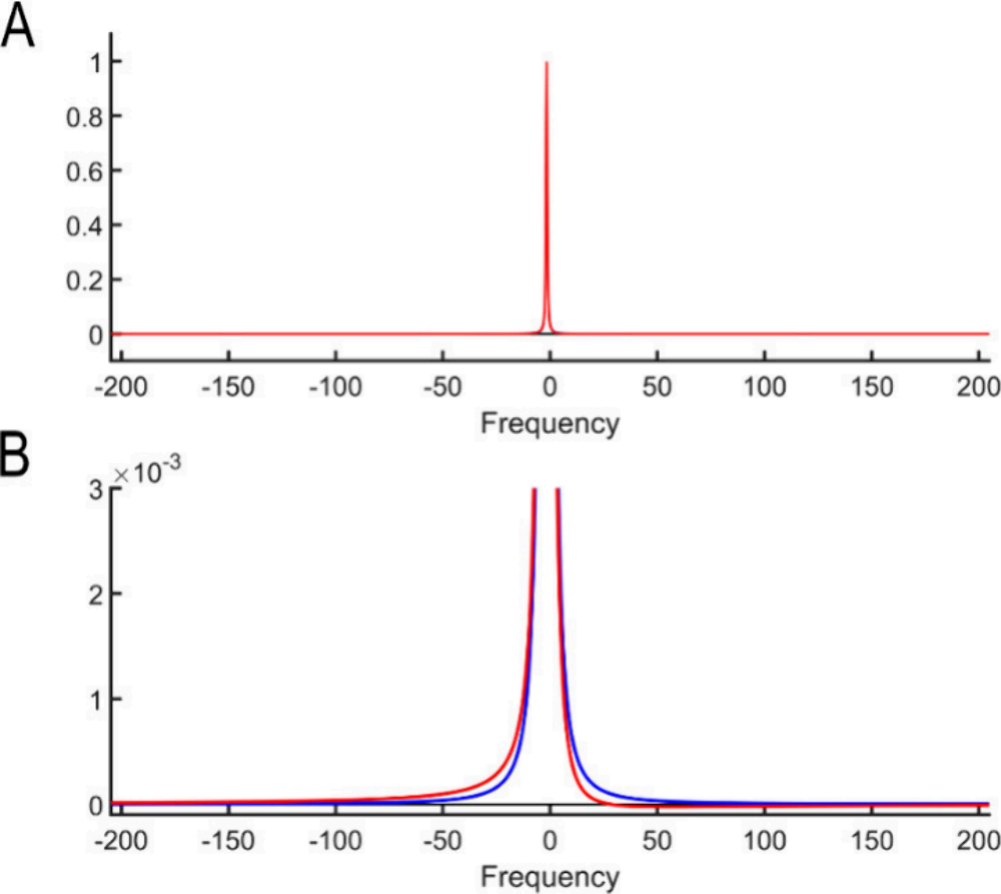
Illustration of a perfectly phased NMR peak (blue), which is 100.0% in absorption mode, and an imperfectly phased peak (red) with a 0.5° phase error. The black horizontal line indicates zero amplitude. (A) At full scale, the imperfectly phased peak is essentially indistinguishable from the correctly phased peak with the two overlapping entirely (within the width of the blue line). (B) As shown in the figure, the phase error becomes apparent when the spectrum is 333-fold enlarged along the *y*-axis.

Recently, attention neural networks, also known as “transformers”, have been introduced with remarkable success to address the long-range memory loss issue in language models (see, e.g. ChatGPT).^20^ Transformers can now effectively capture long-range correlations in time series or spatial data, up to millions of data points away. They were subsequently shown to be suitable for image labeling tasks as well, through a variant known as vision transformers.^24^ Our NMR phase correction method combines a CNN with a vision transformer, a combination frequently used in other fields.^25^ Intuitively, the type of neural network architecture employs the CNN to capture local features and the transformer to capture long-range information. Additionally, the CNN component aids in reducing the length of training samples and, hence, facilitates the training of the transformer. In the original applications of the vision transformer, embeddings of the position of each input picture patch (namely, a small part of the picture of fixed size) are trained concurrently with other parts of the neural network. This approach is crucial as both patch position and patch content need to be interpreted together to infer the meaning of a picture. However, in our automated phase correction neural network, the position of each patch holds distinct information, independent of the spectral data. Consequently, the position information on the input spectrum is encoded using a relative positional encoding scheme that remains invariant to the absolute position while capturing both the sequential order and pairwise distances among elements. We included a global average pooling layer between the transformer and the final, fully connected network, enabling the ANN to process input spectra of arbitrary length, whereas the maximum field of view of the Vision Transformer is 131,072. Our ANN models contain a total of 319,347 training parameters and 192 nontraining parameters. Two independent ANN models were trained to detect zeroth and first order phase errors, referred to as ANN-ph0 and ANN-ph1, respectively. In the phase correction workflow, both networks take the same uncorrected input spectrum and predict whether the PH0 or PH1 component is positive, negative, or zero. The outputs from the ANNs then guide additional zeroth and first order phase corrections until both models indicate no remaining phase error.

Like for the development of DEEP Picker,^17,18^ we opted to use synthetic data instead of true experimental data because DNNs typically require large amounts of training data, which is especially true for transformer networks. Synthetic spectra were generated, based on experimental peak lists from mouse urine and mouse serum samples. They have complex multiplet peak patterns together with a highly concentrated peak region between 3 and 4 ppm with extensive peak overlaps. Data augmentation techniques were employed to enhance the diversity of the training data set by subjecting peaks within the spectra to random modifications, including segment-based peak location rearrangement, removal, and local displacements from their original locations by up to 2000 data points and alterations of the line widths (from 3 to 30 data point) as defined by the full width at half height (FWHH) and peak heights, resulting in a broader distribution of wide, narrow, large and small peaks. Synthetic spectra were also subject to random inversion of the frequency axis because in most experimental NMR spectra peaks reside on the right-hand side of the center of the spectrum, which is usually the solvent line (e.g., water). In addition to these modifications, randomly selected peaks were subjected to further manipulations by introducing overlapping “shoulder” peaks, thereby altering the individual peak profiles to simulate peak line shapes deviating from symmetric Voigt-type line shapes of isolated peaks as observed in experimental spectra. This prevents the network from relying solely on the symmetry of spectral peaks as its target criterion.

Each augmented peak list, generated using the data augmentation techniques described above, was used to synthesize training spectra containing 131,072, 65,536, and 32,768 complex data points. For each spectral length and each peak list, three spectra were generated: one with a positive PH0, one with a negative PH0, and one without any phase error. The absolute PH0 error was randomly sampled between 0.5° and 5.0°. In total, 9,000 training spectra were generated from each of the two experimental peak lists, yielding 18,000 samples across three categories in the PH0 training set. The PH1 training set was constructed similarly, with random first-order phase errors (PH1) ranging from 1° to 10°. Each ANN-ph1 training sample also included a random PH0 error, constrained to the same magnitude as its ANN-ph1 error, to prevent the model from focusing on only a subsection of the spectrum during training. The ANN-ph1 training set also contained 18,000 spectra.

In addition, very broad peaks with FWHH up to 500 data points were included in approximately 30% of the training samples to simulate a spectral background that may be encountered in practice in certain 1D spectra, for example stemming from biomacromolecules such as proteins. To further mimic experimental situations, random Gaussian noise was added to the synthetic spectra. This helps to ensure that the trained model can effectively handle noisy data and still produces robust results. Moreover, a piecewise cubic Hermite interpolating polynomial with five extrema and a maximum amplitude of 0.6% of the largest spectral peak was incorporated. This baseline addition is crucial to prevent the trained model from primarily relying on the presence of negative spectral points or global smoothness of the spectrum to distinguish between incorrectly and correctly phased spectra. Therefore, the model is “forced” to discriminate phase errors based on inherent characteristics of spectral features caused by incorrect phases, thereby achieving more reliable and more robust outcomes.

Training was carried out using TensorFlow v2.19 with GPU support. Our model underwent a total of 400 training episodes, utilizing a min-batch size of 4 and employing an Adam optimizer with a learning rate of 0.0001 and a weight decay rate of 0.00001. Categorical cross-entropy served as the training loss function. The training process spanned approximately 24 h on a workstation computer equipped with 64 GB memory and a single Nvidia RTX 2060TI video card. Given the stochastic nature of neural network training, convergence cannot be guaranteed, especially for complex models like the transformer utilized here. Therefore, a two-step approach was adopted to enhance the success rate of training. The final model was trained based on a pretrained model utilizing a pretraining database. This pretraining database was generated using the same algorithm as the final training database, but with 5–10° phase errors for the ANN-ph0 training set and 5–20° degree phase errors for the ANN-ph1 training set. Consequently, distinguishing unphased spectra from correctly phased ones was made easier for the pretraining database compared to the final database. This strategic approach aimed to increase the likelihood of successful training by providing the model with a foundation built on easier-to-discriminate data before tackling the more challenging task of sometimes subtle phase corrections on the final data set.

All time-domain NMR data sets were processed following a series of standard steps, including apodization, Fourier transformation, and correction of digital filtering of the FID by the Bruker spectrometer. These procedures ensure the removal of any possible PH1 error larger than 360° across the entire spectral width for standard pulse sequences. Subsequently, a two-dimensional grid search for PH0 and PH1 phase correction angles was conducted, with both angles varying from −180° to +180° with 10° increments, based on an entropy minimization algorithm, which typically can bring the remaining PH0 and PH1 phase errors below 10° for spectra containing a baseline, background, and/or many overlapping peaks, as confirmed in our testing. The program then invoked ANN-ph0 and ANN-ph1 to determine the remaining PH0 and PH1 phase errors, applied the additional corrections accordingly, and repeated the process iteratively until both networks predicted no further phase error. Finally, a gradient descent refinement was executed to locate the point where “no-phase-error” prediction scores by both networks were maximized, or until the search step decreased below 0.3°. A neural network inference takes <1 s per spectrum and the total processing time for a typical 64K-spectrum ranges from 5 to 20 s. Figure 2 illustrates the “no-phase-error” prediction scores on a 2D grid, where the *x*- and *y*-axes represent the phase error at the left and right end of the spectrum, respectively, for ANN-ph0 (A) and ANN-ph1 (B). Five representative spectra corresponding to selected points on the 2D surface are shown, with the center corresponding to the phase-error-free spectrum. ANN-ph0 and ANN-ph1 each produces a line on this surface along which the spectra are free of PH0 and PH1 phase errors and achieve maximal scores. The intersection of the two lines corresponds to the correctly phased spectrum, which the automatic phase correction program determines using the outputs from both ANNs as described above. This illustration is provided solely to clarify the method. Importantly, the production program does not rely on an expensive grid search.

**Figure 2:**
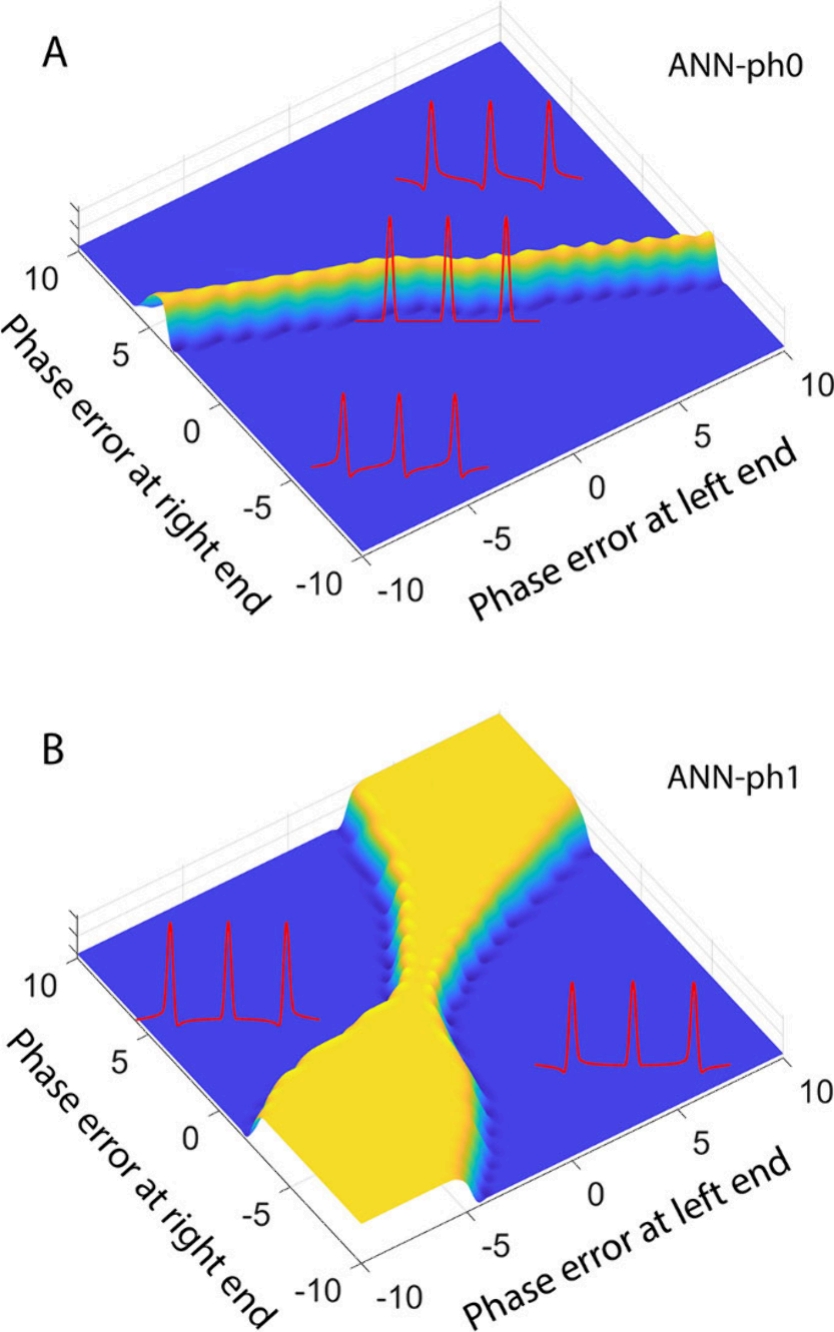
Illustration of “no-phase-error” prediction scores on a 2D grid, where the *x*- and *y*-axes represent the phase errors at the left and right end of the spectrum, respectively, for the tandem vision transformer networks of DEEP Phaser for (A) ANN-ph0 and (B) ANN-ph1. Five representative spectra (red) corresponding to selected points on the grid are shown with the phase-error-free spectrum shown in the center. Panels A and B share the same 2D grid whereby at each grid point both ANN-ph0 and ANN-ph1 receive the same input spectrum with varying phase errors to individually determine the zeroth- and first-order phase prediction scores, which are plotted along the *z*-axis. The yellow regions correspond to the highest prediction scores with the lowest predicted phase errors whereas the blue regions have the lowest prediction scores. The point in the center where both ANN-ph0 and ANN-ph1 have their highest prediction score has zero zeroth- and first-order phase errors corresponding to the optimal global phase correction.

To test the performance of DEEP Phaser for real-world spectra, it was applied to experimental 1D ^1^H spectra of mouse urine, wine, fish oil, DMEM cell growth medium, coffee, K-Ras·GDP protein, and Im7 protein (see the Supporting Information for sample preparation and NMR experimental details). Using a fish oil sample in organic phase (CDCl_3_) as an example, Figure 3 compares a 1D ^1^H spectrum phased using only the entropy minimization method (red) with the spectrum further corrected by DEEP Phaser (blue). The top panel shows the full spectrum, while the bottom panels show two selected peaks at a zoomed-in scale, clearly demonstrating that DEEP Phaser can achieve near-perfect phase correction, whereas the entropy minimization method retains a clearly discernible residual phase error.

**Figure 3:**
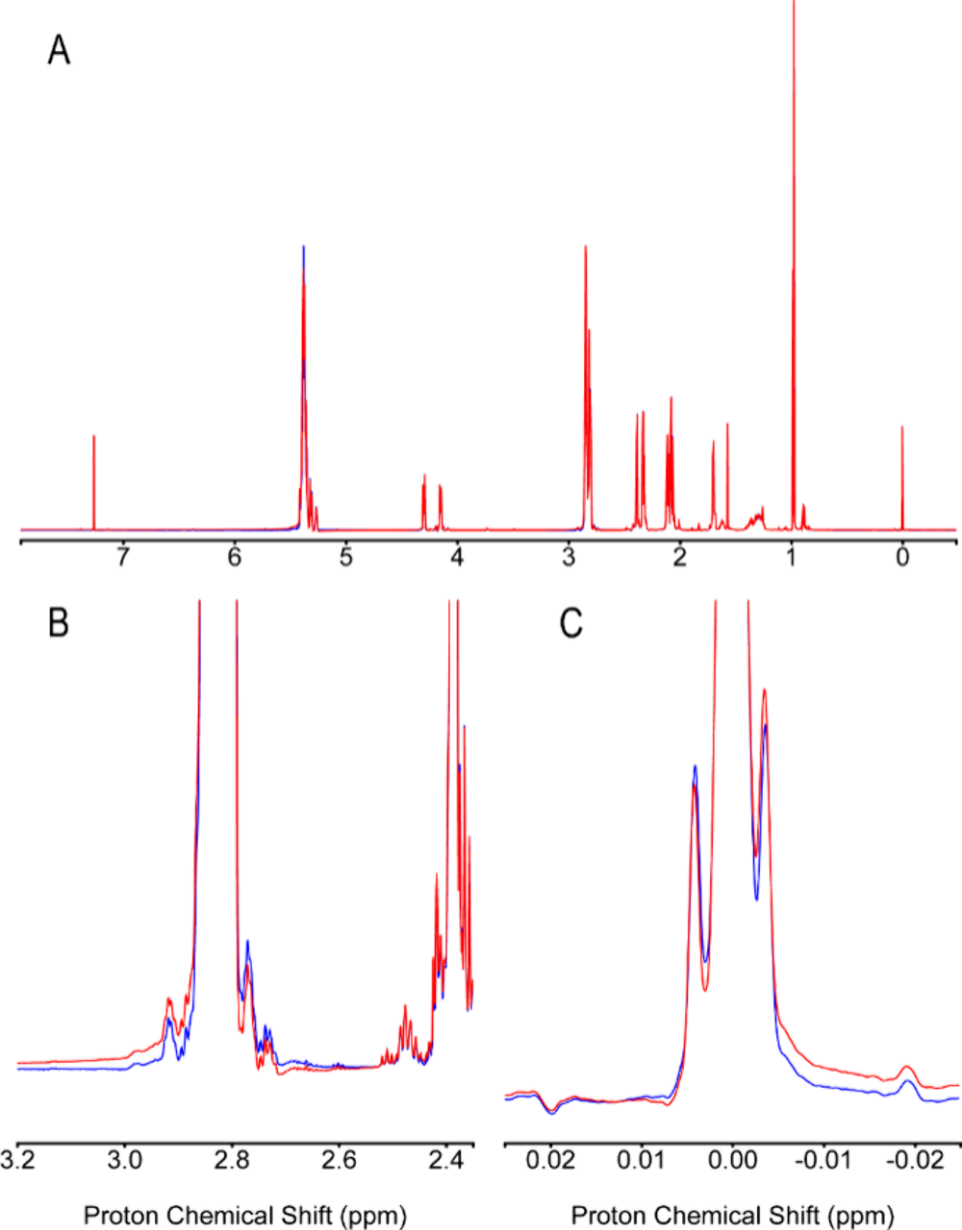
Automated phase-corrected spectra of a fish oil sample in organic solvent (CDCl_3_) measured at 850 MHz obtained using the entropy minimization method (red) and the DEEP Phaser method (blue). Both corrections appear satisfactory at full scale (A), but the zoomed-in regions in (B) and (C) reveal significant differences in performance. For this spectrum, the residual phase error of the entropy minimization method is about 3°.

DEEP Phaser was next applied across a variety of 1D spectra of wine, urine, DMEM, and K-Ras protein at 850 MHz ^1^H frequency demonstrating excellent performance for all spectra (Figure 4). Figure 5 depicts automated phase correction results of mouse serum spectra taken at different magnetic fields at 850, 600, 400, and (benchtop) 80 MHz ^1^H frequency to assess the dependence of its performance on the B_0_-magnetic field strength. In each case, DEEP Phaser achieves essentially perfect phase correction of the input spectra, rendering them suitable for fully quantitative downstream spectral analysis without requiring any additional phasing by an expert spectroscopist. More examples are shown for a spectrum of coffee and Im7 protein in Figure S1. Comparisons with Bruker’s ANN-based phase correction and the recent PD-RAN neural network^19^ are given in Figures S2 and S3 showing clearly better performance by DEEP Phaser.

**Figure 4:**
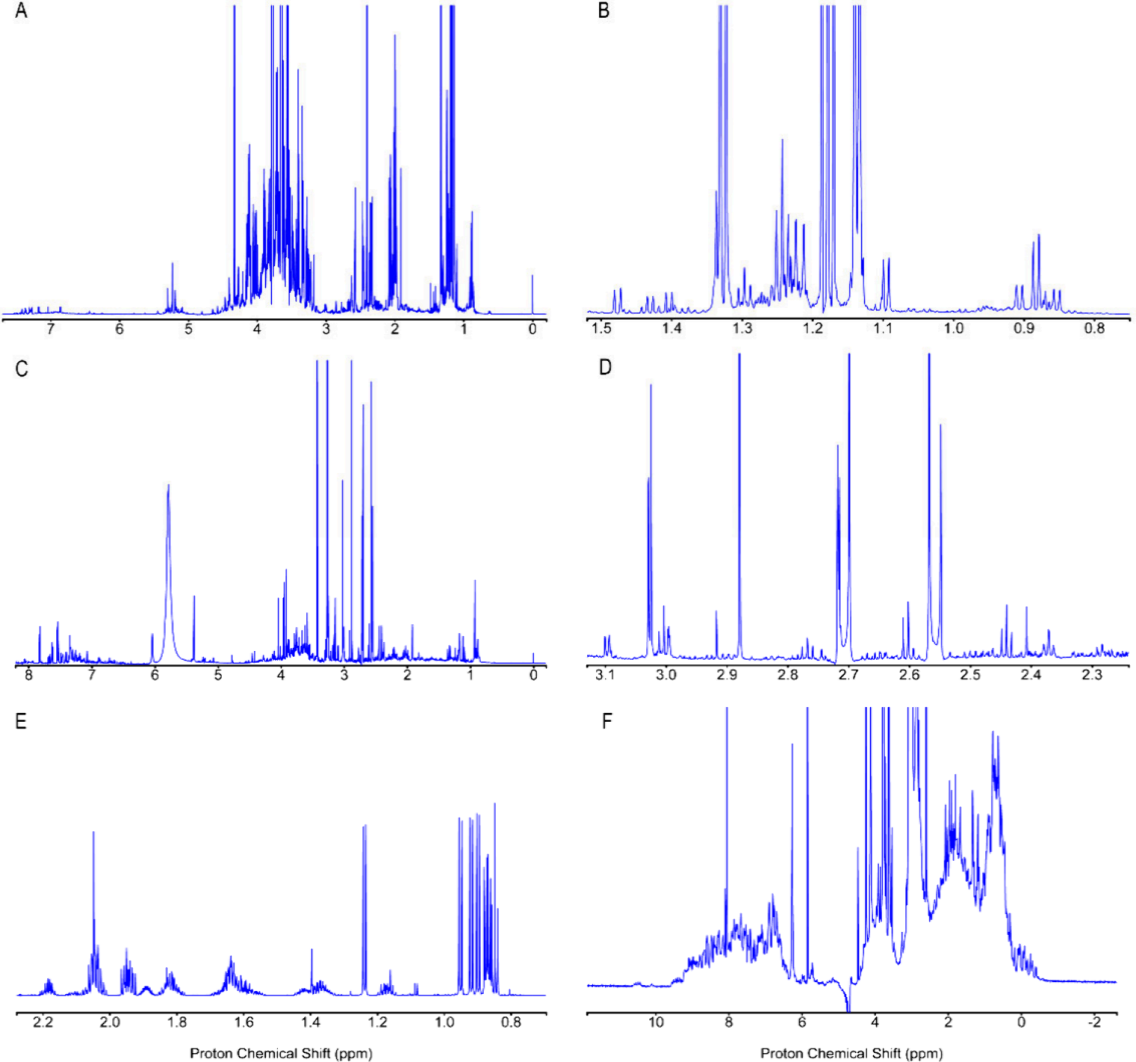
Automated phase-corrected spectra obtained using the DEEP Phaser method for (A,B) wine, (C,D) mouse urine, (E) DMEM, and (F) K-Ras·GDP protein. Panels (B,D) show zoomed-in regions of the full spectra (A,C).

**Figure 5:**
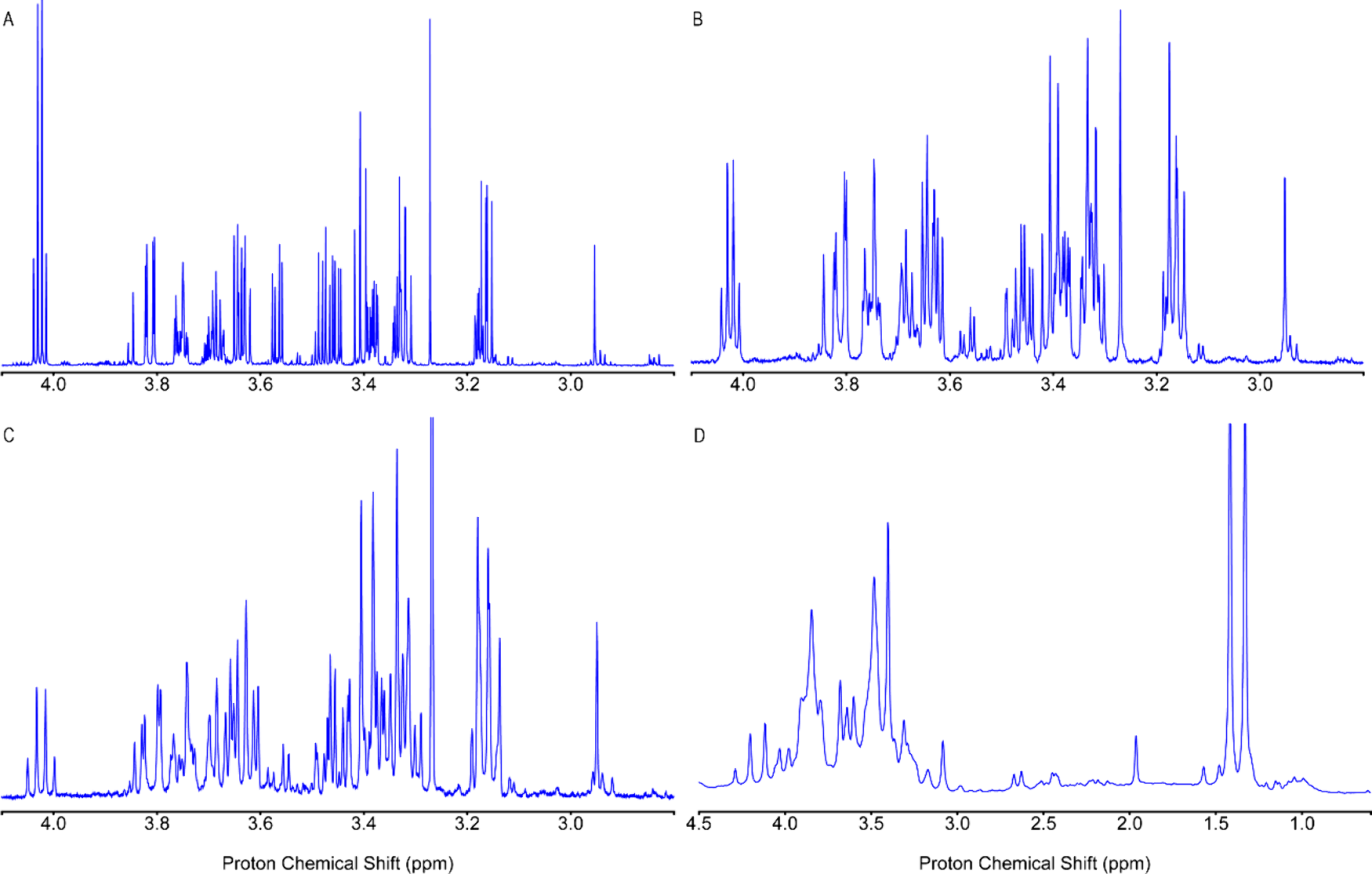
Phase-corrected spectra obtained using the DEEP Phaser method for mouse serum samples in the aqueous phase acquired at (A) 850 MHz, (B) 600 MHz, (C) 400 MHz, and (D) 80 MHz. Except for (D), all panels display the same ppm range.

The DEEP Phaser ANN introduced here for fully automated 1D spectral phase correction performs very well within its trained domain, accurately identifying and correcting PH0 and PH1 errors across a wide range of solution NMR spectra, as shown in Figures 4 and 5. Its validity is naturally limited to the types of spectra and error ranges included in the training set. The training set comprises spectra with lengths from 32K to 128 K and peak widths from 3 to 30 data points (FWHM), covering most typical 1D ^1^H NMR experiments. Approximately 30% of the training samples also included broader peaks, up to 300 data points, to account for larger molecules. In our test, the method requires of the order of 15 or more peaks to be present in the spectrum. Applying the network to spectra with features or phase errors outside this domain is not guaranteed to yield reliable results. In addition, our ANN models are specifically designed to detect only PH0 and PH1 phase errors. For NMR experiments with significant third and higher-order phase errors, the models will not be able to achieve optimal phase correction for all peaks in a spectrum.

While the ANN largely operates as a “black box”, DEEP Phaser likely identifies phase errors by evaluating peak symmetry and the uniform decay of signal tails. This is supported by the self-attention maps in Figures S5 and S6, which highlight the specific spectral regions prioritized by the model. For NMR spectra with poor shimming and resulting peak distortions, the model can recognize the abnormal shape and avoid the use of peak symmetry as a criterion for phase error detection, similar to how it handles regions with heavy peak overlap. On the other hand, for very subtle distortions that are difficult to detect for the human eye, the trained ANN will attempt to balance peak symmetry and equal tail decay to produce the best possible phased spectrum.

The earliest data points of the detected FID are usually missing or distorted due to the finite receiver dead time, which can cause global, low-amplitude baseline distortions or “wiggles” in the resulting frequency-domain spectrum. To mitigate such effects, spectrometer manufacturers apply a correction scheme involving digital filtering, which is often proprietary. The goal of this process is to cancel the baseline distortion, ideally producing a perfectly flat baseline at a zero-intensity level. In our experience, however, perfect baseline correction is not guaranteed, and its performance varies in a spectrum-dependent manner. While it is tempting to phase the spectrum to achieve the flattest possible baseline, including minimizing distortions at the spectral edges, this approach may conflict with the optimal phasing required for individual peaks. DEEP Phaser prioritizes correct phase correction of all individual peaks over achieving a flat baseline. Any resulting baseline artifacts, including distortions at the spectral edges, can then be addressed or removed in a subsequent step after the phase correction is complete. For this purpose, we implemented a statistical-based baseline correction method by Xi and Rocke^26^ and incorporated it into our automated phase correction pipeline. The baseline correction can be adjusted to the specific needs for a certain spectrum, for example, to effectively eliminate the broad protein background of certain metabolomics samples as demonstrated in Figure S4. DEEP Phaser with baseline and background correction has been implemented in the public domain COLMARvista software^23^ and COLMAR web servers,^21,22,27^ DEEP Phaser can be adopted for the phasing of 2D and higher-dimensional NMR spectra by applying it to individual 1D cross sections or projections.

In summary, we presented the machine learning-based automated NMR phase correction algorithm “DEEP Phaser”, leveraging the combined power of classical convolutional neural networks and transformer networks. While CNNs excel at local pattern recognition, transformers, owing to their attention mechanism, can effectively identify even very small spectral phase errors by simultaneously analyzing spectra over many data points and a large spectral range. Our phase correction model underwent rigorous testing across a diverse range of experimental 1D NMR spectra, demonstrating excellent accuracy. DEEP Phaser can be adopted for the phasing of 2D and higher-dimensional NMR spectra by applying it to individual 1D cross sections or projections. The achieved phase correction accuracy and robustness for complex NMR spectra enables the integration of DEEP Phaser in fully automated NMR workflows overcoming the need for human intervention for this small, but crucial data processing step.

## Supplementary Material



## Data Availability

Automated phase correction algorithm is now included in our DEEP1d and COLMAR1d Web servers at https://colmar.cloud and our COLMARvista processing/visualization software at https://vista.colmar.cloud using the TensorFlow.js library. The repository (https://github.com/lidawei1975/phase_1d) includes MATLAB scripts for synthetic data generation and Python code for model training and definition.

## Notes

Details about sample preparation and NMR experiments; more phase correction examples, including mouse serum spectrum with protein background and its algorithmic removal and coffee; comparisons with latest state-of-the-art Bruker Topspin machine learning–based method and residual attention network-based PD-RAN; self-attention maps for ANN-ph0 and ANN-ph1 (PDF)
