# Supplementary material for: DEEP Phaser: A Deep Learning Tandem Vision Transformer for Fully Automated NMR Phase Correction
Source: J Phys Chem Lett. 2026 May 4;17(19):5520–6. doi: 10.1021/acs.jpclett.6c00770 (PMC13181785; doi:10.1021/acs.jpclett.6c00770)
Supplement: Supplementary file 1 [file jz6c00770_si_001.pdf]

Supplementary Information

DEEP Phaser: A Deep Learning Tandem Vision Transformer for Fully Automated NMR Phase Correction

Da-Wei Li,[1*] Lei Bruschweiler-Li,[1] Kyungsuh Lee,[1] and Rafael Brüschweiler [1,2*]

[1]Department of Chemistry and Biochemistry, The Ohio State University, Columbus, Ohio 43210, USA

[2]Department of Biological Chemistry and Pharmacology, The Ohio State University, Columbus, Ohio 43210, USA

Content

Sample preparation; NMR experiments; protocols; additional NMR spectra, including spectrum with protein background, obtained with DEEP Phaser; a performance comparison of DEEP Phaser with the latest deep neural networks implemented in Topspin by Bruker and the PD-RAN method; self-attention maps for PH0 and PH1.

NMR sample preparation

This project used a variety of different samples to test the performance of the DEEP Phaser software, including mouse urine, wine, fish oil, DMEM cell growth medium, coffee, K-Ras·GDP, and Im7. Samples were prepared as follows:

The mouse urine, wine, DMEM, K-Ras·GDP, and Im7 samples were prepared as described previously [S1,S2,S3] and measured in aqueous buffer with 5 - 10% D_2O. For the mouse serum sample, 300 μL aliquot of mouse serum was solvent extracted with chilled water, methanol, and chloroform at 1:1:1 ratio and the polar phase extract was then lyophilized. Lyophilized extracts were resuspended in D_2O NMR buffer (50 mM sodium phosphate buffer, pH 7.4), subjected to ultrafiltration (Pall) for further protein removal followed by the addition of 0.1 mM DSS. The protein-containing serum sample was prepared as above but without the ultrafiltration step.

The fish oil sample was obtained from soft gel capsules (Sports Research, Triple Strength Omega-3 Fish Oil; dietary supplement). An aliquot of the fish oil was diluted in chloroform-d (99.8% deuterated) containing 0.03% (v/v) tetramethylsilane (TMS) (VWR Chemicals BDH) to a final fish oil content of 1.2% (v/v). The final concentration of TMS in the sample was 2.2 mM. The NMR tube was sealed with a PTFE cap to minimize solvent evaporation prior to and during NMR measurements.

For the coffee sample, espresso coffee was subject to solvent extraction with chilled methanol and chloroform at 1:1:1 ratio; the aqueous phase was recovered and lyophilized. The lyophilized powder was resuspended in 50 mM sodium phosphate buffer pH 7.4 containing 10% D_2O and the pH was further fine-tuned with 1 M NaOH to 7.4, DSS was added to be at 0.1 mM final concentration.

NMR experiments

NMR 1D ^{1}H experiments were performed at 850 MHz (Bruker Avance III with TCI cryoprobe), 600 MHz (Bruker Avance III with BBFO probe), 400 MHz (Bruker Neo with BBFO probe), and 80 MHz (Bruker Fourier 80 benchtop without pulsed field gradients) NMR spectrometers. The acquisition times ranged from 3 – 6 seconds with 64k – 128k complex points. This was followed by 2 or 4-fold zero-filling, apodization with a 2π-Kaiser window using COLMARvista,[22] but without phase and baseline correction. The Bruker pulse sequence "noesygppr1d" was used for all data collected from 400 MHz to 850 MHz, whereas the sequence "noesypr1d" was used at 80 MHz.

References

[S1] Li, D. W.; Cabrera Allpas, R.; Choo, M.; Bruschweiler-Li, L.; Hansen, A. L.; Brüschweiler, R., COLMAR1d: A Web Server for Automated, Quantitative One-Dimensional Nuclear Magnetic Resonance-Based Metabolomics at Arbitrary Magnetic Fields. *Anal Chem* **2024**, *96* (43), 17174-17183.

[S2] Hansen, A. L.; Xiang, X.; Yuan, C.; Bruschweiler-Li, L.; Brüschweiler, R., Excited-state observation of active K-Ras reveals differential structural dynamics of wild-type versus oncogenic G12D and G12C mutants. *Nat Struct Mol Biol* **2023**, *30* (10), 1446-1455.

[S3] Xie, M., Yu, L., Bruschweiler-Li, L., Xiang, X., Hansen, A.L. and Brüschweiler, R., 2019. Functional protein dynamics on uncharted time scales detected by nanoparticle-assisted NMR spin relaxation. *Science advances* **2019**, *5*(8), p.eaax5560.

[S4] Zhao, C.; Chen, G.; Liu, C.; Li, Z.; Zhao, J.; Sun, P.; Chu, H.; Zhou, X.; Liu, M.; Song, P.; Bao, Q.; Liu, C., Phase Model-Driven Deep Learning for Robust Phase Correction in High-Throughput NMR-Based Metabolomics. *J Phys Chem Lett* **2026,** *17* (4), 1245-1253.

[S5] Xi, Y.; Rocke, D. M., Baseline correction for NMR spectroscopic metabolomics data analysis. *Bmc Bioinformatics* **2008,** *9*, 324.

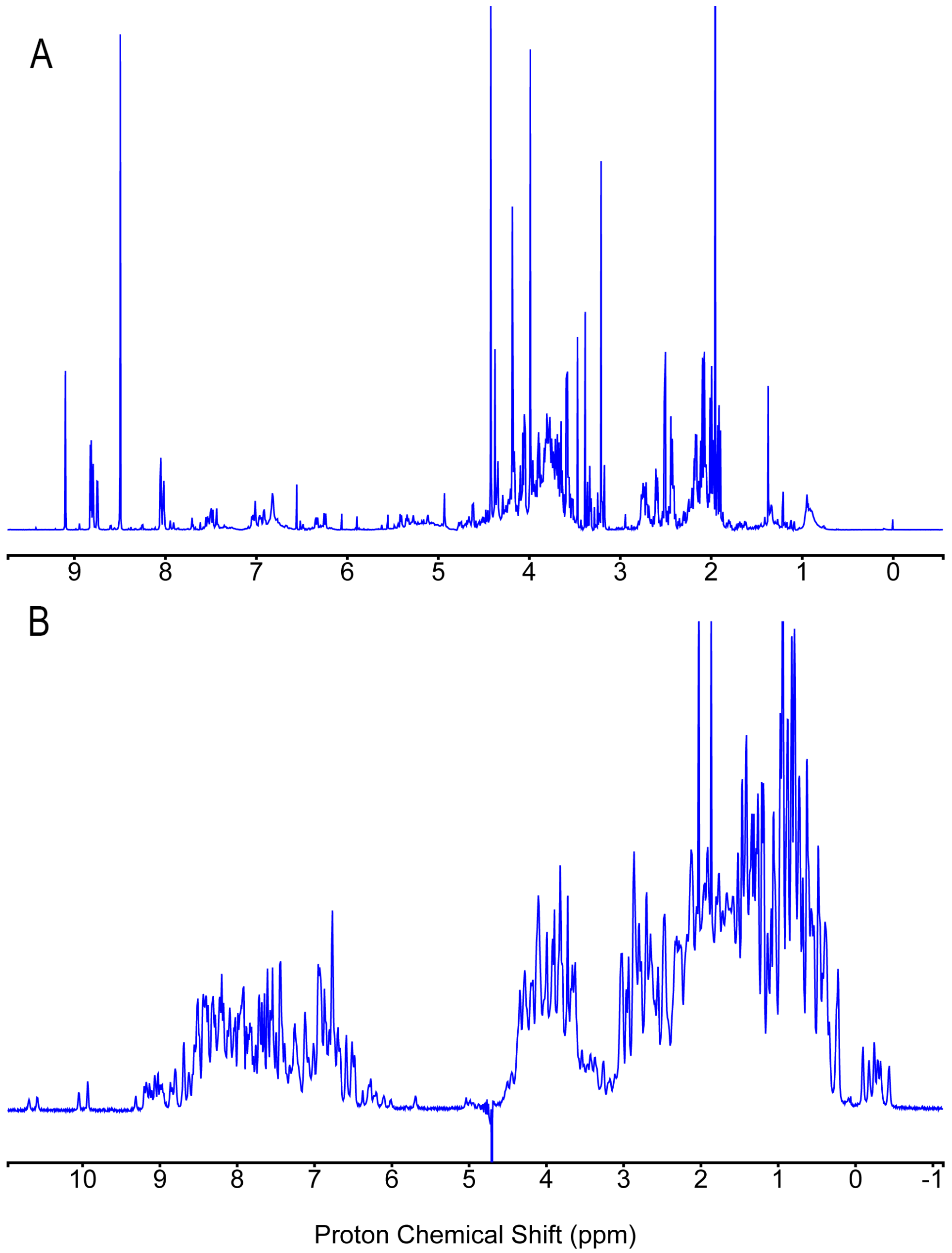

Figure S1. Phase-corrected spectra obtained using the DEEP Phaser method for (A) coffee and (B) protein Im7. All spectra were acquired in aqueous solution at 850 MHz ^{1}H frequency.

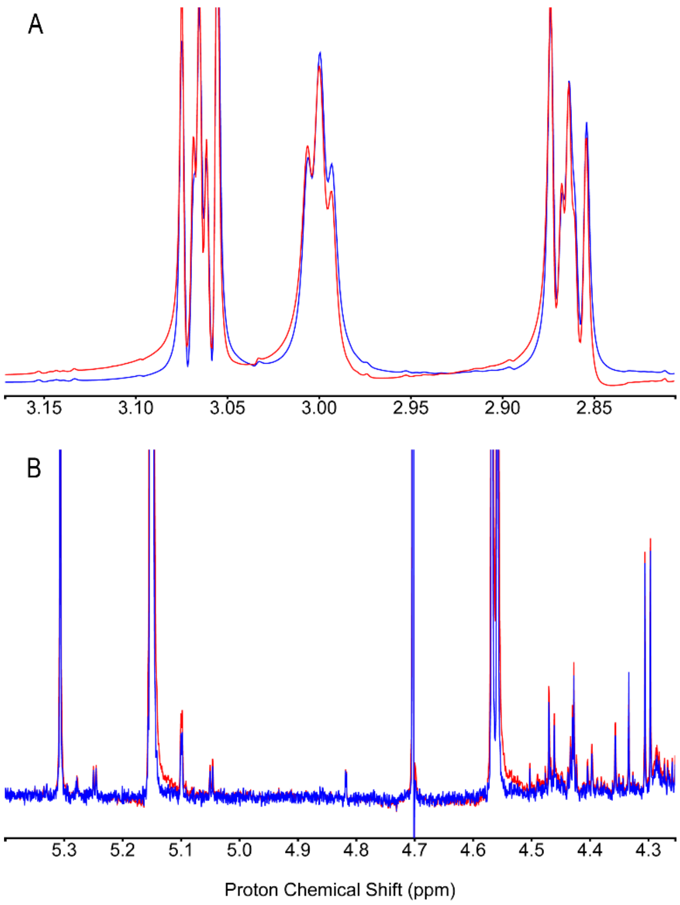

Figure S2. Selected zoomed-in regions of phased spectra for (A) K-Ras·GDP and (B) serum, acquired at 850 MHz and processed using the DEEP Phaser method (blue) and the latest Bruker machine-learning based method implemented in Topspin (red, using TopSpin command "apbk -f"). DEEP Phaser shows better performance, although both methods may be sufficient for many practical applications.

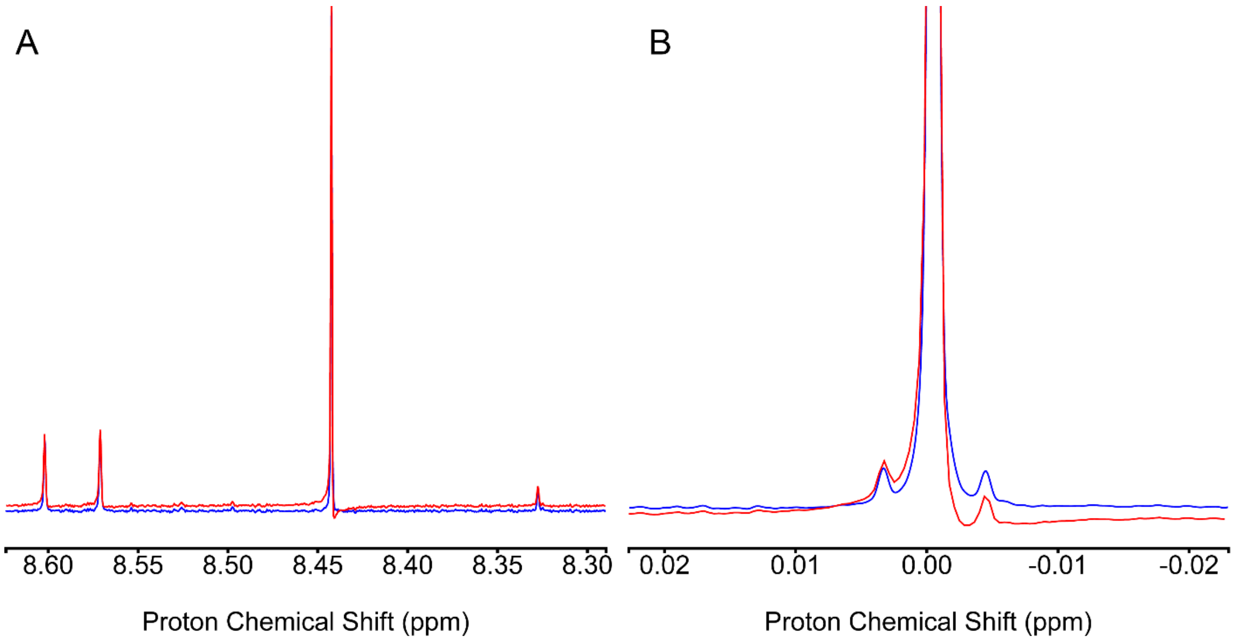

Figure S3. Selected zoomed-in regions of phased mouse serum spectra processed with the PD-RAN neural network (red) [S4] in comparison with DEEP Phaser (blue). In contrast to the blue spectrum, a phase error in the red spectrum of several degrees is well visible. The performance of PD-RAN appears to depend on the type of sample.

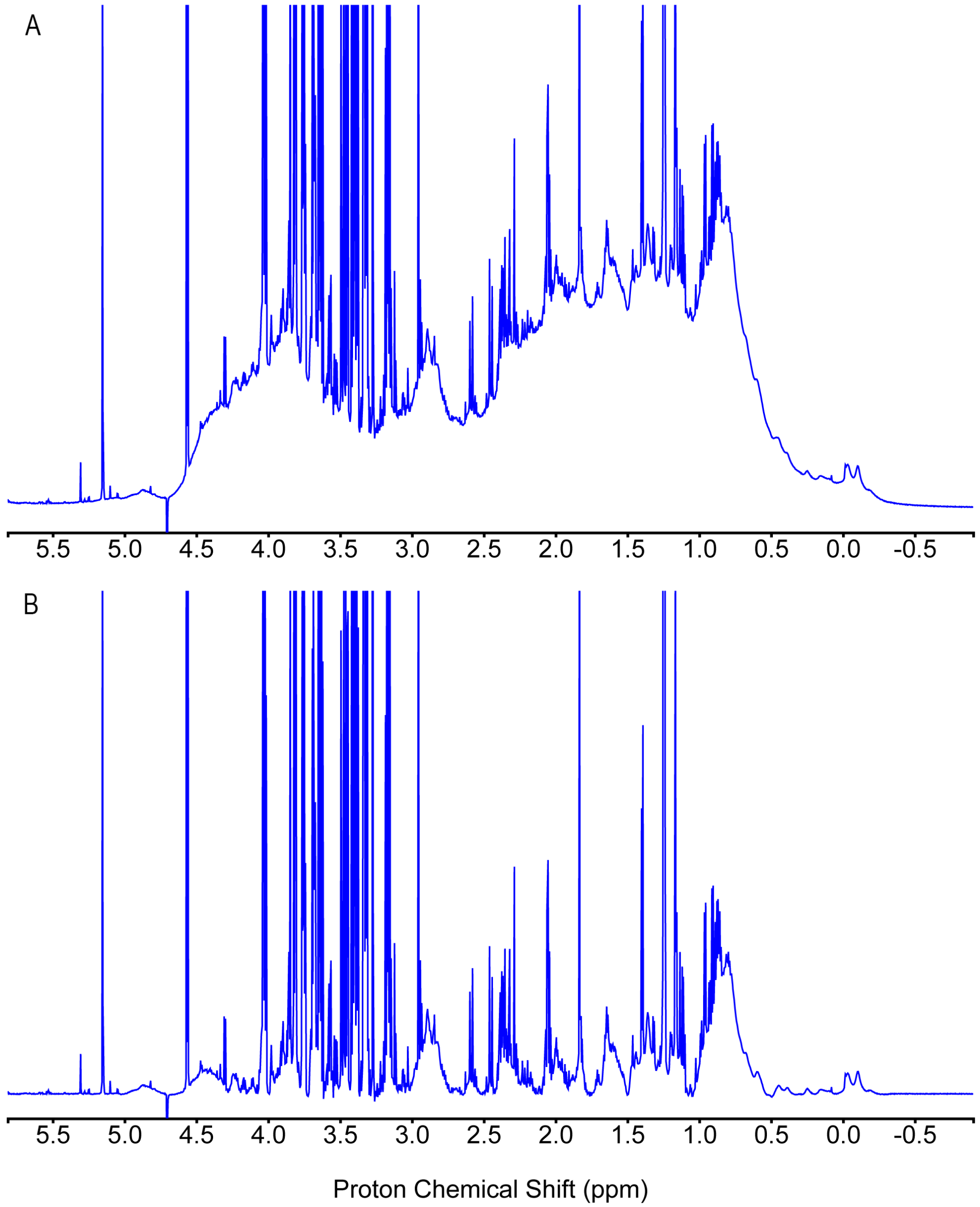

Figure S4. Phase-corrected spectra obtained using the DEEP Phaser method for mouse serum sample with protein present (A) before and (B) after background removal using the parametric baseline removal model by Xi and Rocke [S5].

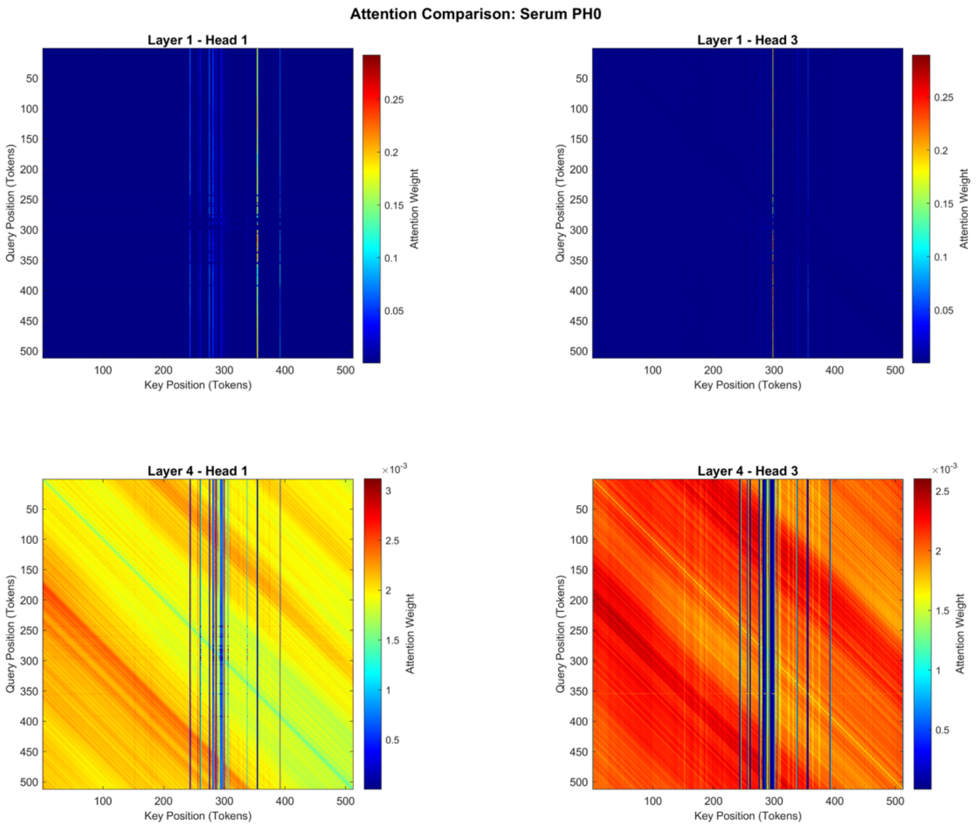

Figure S5. Visualization of self-attention map for PH0 at the example of a mouse serum spectrum (64K points) for 2 out of 4 heads (Head 1 and 3) of the 1st and 4th layer. The initial layer (top two panels) exhibits vertical attention strips with high relative weights, indicating the identification of global spectral anchors and large peaks for normalization. In subsequent layers (bottom two panels), the mechanism transitions toward tilted diagonal strips with significantly lower maximum weights, suggesting a transition from local peak selection to a distributed integration strategy where the network models subtle relative positional dependencies and linear phase gradients across the entire spectrum. For PH0, the model prioritizes absolute spectral references before introducing relational fine-tuning, as the frequency-independent nature of zero-order phase makes absolute identification more critical than the relative tracking required for PH1.

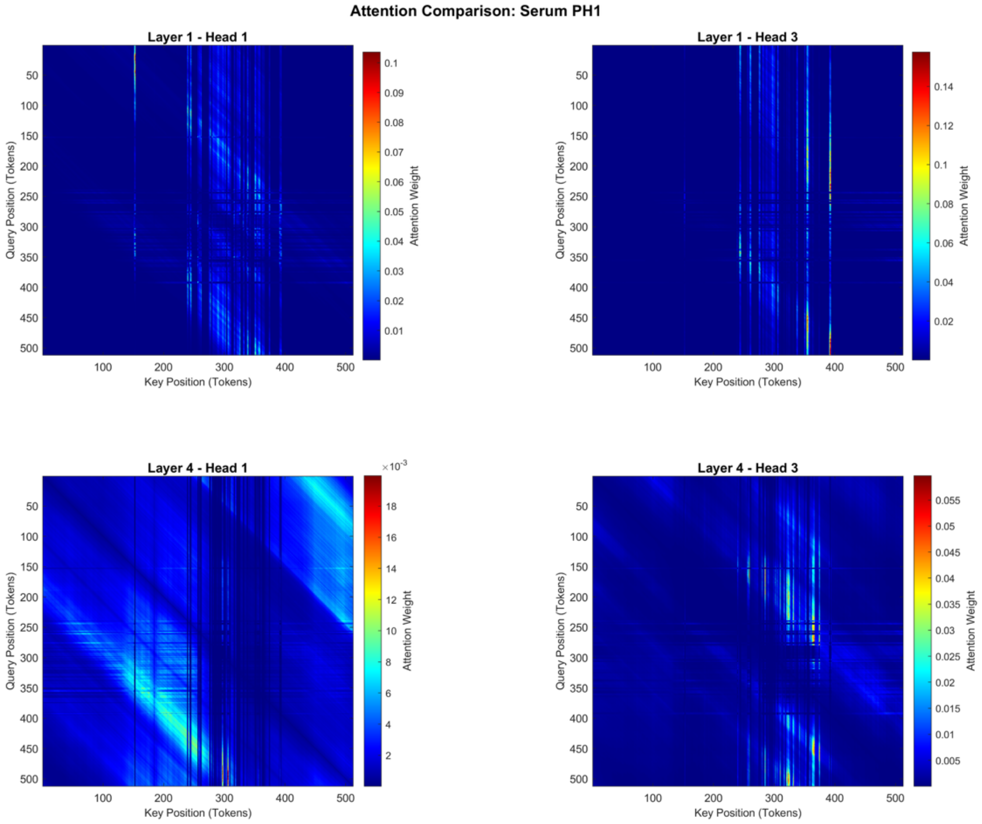

Figure S6. Visualization of self-attention map for PH1 at the example of a mouse serum spectrum (64K points) for 2 out of 4 heads (Head 1 and 3) of the 1st and 4th layer. These layers exhibit vertical attention strips of varying weights, indicating the identification of global spectral anchors and prominent peaks for normalization. Additionally, the tilted strips reflect the frequency-dependent nature of the first-order phase error, which is consistent with the relative positional encoding implemented in the network.
